# Prescribing cascades of antigout medications from thiazide diuretics in gout-naïve hypertensive adults receiving first-line pharmacological management

**DOI:** 10.1038/s41598-024-58153-0

**Published:** 2024-03-28

**Authors:** Shang-Yeh Lu, Hsing-Yu Hsu, Yow-Wen Hsieh, Chiung-Ray Lu, Hsin-Yi Huang, Shih-Sheng Chang

**Affiliations:** 1 Division of Cardiology, Department of Internal Medicine, China Medical University Hospital, No. 2, Yuh-Der Road, Taichung, Taiwan; 2 Graduate Institute of Biomedical Sciences, China Medical University, No. 91, Xueshi Rd., Taichung, Taiwan; 3 Department of Pharmacy, China Medical University Hospital, No. 2, Yuder Rd., Taichung, Taiwan; 4 School of Pharmacy, College of Pharmacy, China Medical University, No. 100, Sec. 1, Jingmao Rd., Taichung, Taiwan; 5 School of Medicine, College of Medicine, China Medical University, No. 100, Sec. 1, Jingmao Rd., Taichung City, Taiwan; 6 Graduate Institute of Clinical Pharmacy, College of Medicine, National Taiwan University, No.33, Linsen S. Rd., Taipei, Taiwan

**Keywords:** Prescribing cascade, Thiazide, Hypertension, Gout, Cardiology, Hypertension

## Abstract

Prescribing cascade is a significant clinical problem but is often overlooked. We explore the incidence of the prescribing cascades of antigout medications related to thiazide treatment in gout-naïve hypertensive adults newly exposed to the pharmacological treatment. This population-based, retrospective cohort study used the Taiwan National Health Insurance Registry Database. Gout-naïve hypertensive adults who were newly dispensed first-line antihypertensive drugs between January 1, 2000, and December 31, 2016, were enrolled. Patients were divided into the thiazide group (n = 4192) and the non-thiazide group (n = 81,083). The non-thiazide group included patients who received an angiotensin-converting enzyme inhibitor, angiotensin II receptor blocker, calcium channel blocker, or beta-blocker. The study utilized propensity score matching and multivariable Cox regression models to investigate the prescribing cascade of antigout agents following antihypertensive treatment, adjusting for factors like age, sex, comorbidities, and concurrent medications. After propensity score matching, each group consisted of 4045 patients, with the thiazide group exhibiting a higher risk of being prescribed antigout medications across different time intervals post-treatment initiation. Specifically, adjusted hazard ratios (aHRs) for the thiazide group were 2.23, 2.07, and 2.41 for < 30 days, 31–180 days, and > 180 days, respectively, indicating a sustained and significant risk over time. Comparative analyses revealed thiazide diuretics were associated with a higher risk of antigout medication prescriptions compared to other antihypertensive classes, particularly evident after 180 days. Subgroup analyses across various demographics and comorbidities consistently showed an increased risk in the thiazide cohort. Gout-naïve hypertensive adults newly dispensed thiazide had a higher risk of subsequently adding antigout agents than those taking other first-line antihypertensive medications. The awareness and interruption of these prescribing cascades are critical to improving patient safety.

## Introduction

The prescribing cascade is an important issue in clinical practice. It refers to the phenomenon wherein the adverse effects of a medication prescribed for a specific disease are misinterpreted as a new medical problem and treated by other drugs by the same doctor or other doctors^1–3^. The geriatric population is at a particularly high risk of prescribing cascades due to comorbidities and the subsequent use of multiple medications. Further, the side effects of the subsequently prescribed agents and the drug–drug interactions can lead to adverse outcomes, including urgent hospitalization^4^, increased hospital stay^5^, and higher medical costs^6^.

Hypertension is a highly prevalent chronic disease. The following five classes of drugs are typically used as the first-line pharmacological therapy for hypertension: thiazide diuretics, beta-blockers (BBs), calcium channel blockers (CCBs), angiotensin-converting enzyme inhibitors (ACEIs), or angiotensin II receptor blockers (ARBs)^7,8^. Thiazide diuretics are an effective and low-cost choice^9,10^. However, these drugs can cause electrolyte imbalance, hyperlipidemia, and elevated serum uric acid levels by decreasing the excretion of uric acid via the kidney^11,12^. Thiazide diuretics influence the serum uric acid level by altering the mechanism of secretion and uptake of uric acid in the proximal renal tubule cells. Furthermore, thiazide diuretics also lead to salt and volume depletion, which enhances uric acid reabsorption^13^. Nonetheless, a certain proportion of hypertensive patients require treatment with thiazide or thiazide-like diuretics to attain blood pressure control^14^. Other than these populations, there is no evidence to show that thiazide is mandatory for hypertension management or is more effective than other antihypertensive agents^8,15,16^. In addition, different classes of antihypertensive medications have also been reported to influence the serum uric acid level^12,17^. BBs and some ARBs were reported to increase the uric acid concentration, while ACEIs and CCBs were shown to have no effect or to decrease serum uric acid^18^.

Urate-lowering agents, e.g., benzbromarone, allopurinol, febuxostat, colchicine, and non-steroid anti-inflammatory drugs (NSAIDs), are frequently used for clinically significant hyperuricemia or acute exacerbation of gout. These drugs can cause specific adverse effects, such as acute kidney injury, gastrointestinal bleeding, allergy, and drug–drug interactions^5,19^. In this context, management of hyperuricemia with antigout medication instead of discontinuing the potential offending drugs can expose the patient to unnecessary risks, increase medical costs, as well as aggravate the pill burden. Furthermore, some antigout agents can cause potentially fatal adverse effects, such as allopurinol-related Stevens-Johnson syndrome or toxic epidermal necrolysis^19^.

There is a paucity of studies exploring the association between the use of thiazide diuretics and prescribing cascades of antigout medicine in gout-naïve hypertensive adults who were newly exposed to first-line antihypertensive medications. We conducted a nationwide retrospective cohort study to examine whether thiazide diuretics in hypertension management are associated with an increased prescribing cascade of antigout agents among first-line antihypertensive medications.

## Materials and methods

### Data sources

This was a population-based retrospective cohort study using the database of the Taiwan National Health Insurance (NHI) program. The NHI program covers more than 99.6% of Taiwan’s 23.74 million residents; the claims data from this program are released as the National Health Insurance Registry Database (NHIRD). The NHIRD contains claims data, including a registry of beneficiaries, records of inpatient and outpatient care, drug prescriptions, and other medical services, and these data are renewed annually. The data is based on the International Classification of Diseases, Ninth & Tenth Revision, Clinical Modification (ICD-9-CM; ICD-10-CM). Medication is recorded with an ATC code. The codes for diagnosis and prescription used in the present study are listed in the supplemental tables. The longitudinal generation tracking database (LGTD) comprises randomly selected two million beneficiaries from the NHIRD from 2000 to 2017. This study used the LGTD for analysis. Before the data are released for research, the Taiwan government implements privacy protection for insured individuals, removes the original identification numbers, and provides an encoded serial number for insured individuals to link their claims data. This study was approved by the Ethics Review Board of China Medical University (CMUH109-REC2-031), which waived the requirement for informed consent. The study used anonymized data from the Taiwan NHIRD. The research design and data management strictly adhered to ethical principles and IRB guidelines.

### Study cohort

**Figure 1:**
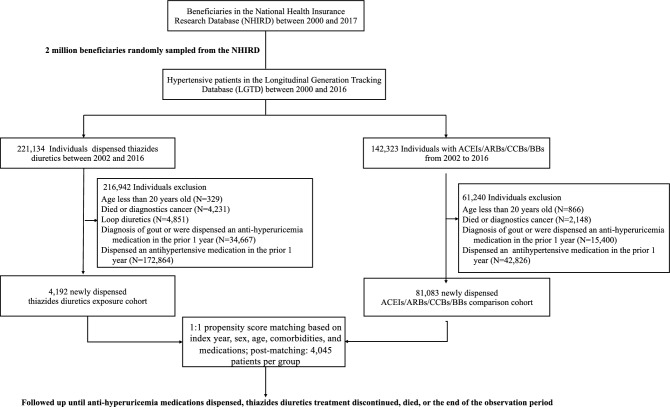
Flowchart of patient enrollment. *ACEIs* angiotensin-converting enzyme inhibitors, *ARBs* angiotensin II receptor blockers, *CCBs* calcium channel blockers, *BBs* beta-blockers.

Adult patients with hypertension who were treated with first-line antihypertensive medications (e.g., ACEIs, ARBs, BBs, CCBs, thiazide) were included in this study (Table S1). Hypertension was determined based on the diagnosis of hospital admission or at least three times of outpatient visits from January 1, 2000, to December 31, 2017, in the LGTD (Table S2). We then formed two cohorts from the beneficiaries: one of 221,134 individuals who have dispensed thiazide diuretics and another of 142,323 patients who were treated with ACEIs, ARBs, BBs, or CCBs between 2002 and 2016. From these groups, we excluded individuals under the age of 20, those who had died before the index date, patients with a prior diagnosis of cancer, those with a history of gout or who had been prescribed antigout medications in the year preceding the index date, and patients who had used any antihypertensive drugs in the year before the index date. For the thiazide cohort, we further excluded individuals being prescribed loop diuretics. This process yielded a thiazide cohort with 4192 patients and a non-thiazide cohort with 81,083 patients. Both cohorts were then matched in a 1:1 ratio based on propensity scores, which considered age, sex, hypertension duration, comorbidities, and medications, resulting in groups of 4045 patients each (Fig. 1). The non-thiazide group was further categorized into subgroups based on their initial medication—ACEIs, ARBs, BBs, or CCBs. It's important to note that while the non-thiazide group had no thiazide exposure, the thiazide cohort may have received ACEIs, ARBs, BBs, or CCBs treatments following their thiazide regimen. The first dispensing date of the antihypertensive medications was defined as the index date. New exposure is defined as the initial administration of a target antihypertensive medication, either thiazide diuretics or from classes including ACEIs, ARBs, BBs, or CCBs, and also applies to individuals who have not received any antihypertensive medication in the year prior to starting treatment with the target drug. Furthermore, a hospital-based study showed that drugs are a major cause of elevated serum uric acid concentration^20^. Therefore, for further adjustment, we also extracted the data of concurrently used related medications that may interfere with uric acid levels, including antituberculosis agents, immune-suppressive agents, nicotinic acid, and aspirin (Table S1)^13^.

### Outcome measurement

The primary outcome was the prescribing cascade of antigout medication, which was defined as the dispensing of one of the predefined antigout agents to the patient during the follow-up period after the index date. We defined the antigout agents as allopurinol, febuxostat, benzbromarone, and colchicine (Table S1). To better understand the temporal trend of prescribing cascade, patients were observed for at least 180 days since the index date. The follow-up ended when the first subsequent dispensing of antigout drugs occurred, antihypertensive medicine was discontinued, patients died, or the observation period ended, whichever came first.

### Statistical analysis

In this study, patients were matched on a 1:1 basis using propensity score matching to balance important variables, including age, gender, duration of hypertension, and comorbidities, between the thiazide and non-thiazide cohorts. Table 1 presents the baseline characteristics and comparisons of the original cohort and the post-matching groups, employing the Student's t-test for continuous variables and the Chi-squared test for categorical variables. Multivariable Cox proportional hazards regression models were used to determine whether thiazide treatment was independently associated with the prescribing cascade of antigout agents. The models were adjusted for potential confounding factors, including age, sex, comorbidities, and concurrent relevant medications, and the results were reported as hazard ratios (HRs) and 95% confidence intervals (CIs)^13^. Furthermore, potential interaction effects between thiazide use and these covariates were assessed, and statistically significant interactions were integrated into the models to examine the differential impact of thiazide use. In addition to the primary analysis, Supplementary Table S3 presents a separate exploratory analysis detailing both crude and adjusted hazard ratios for each covariate. This table offers a comprehensive view of the associations of each covariate with the risk of being prescribed antigout medications. To obtain more robust results, subgroup analysis was performed to evaluate the risk of adding antigout drugs after using thiazides for hypertension management. All the parameters in the subgroup analyses were defined a priori. We performed stratified analyses by sex, given that men have been reported as being more likely than women to experience hyperuricemia^21^. In addition, subgroup analysis was performed according to chronic kidney disease (CKD), diabetes mellitus (DM), and coronary artery disease (CAD), which are believed to be linked to the progression of hyperuricemia^17,22^. Moreover, in previous studies, other first-line antihypertensive medications were also found to influence the plasma uric acid level^18^; therefore, we also investigated the prescription of antigout medicines among each category of antihypertensive medicine, i.e., ACEIs, ARBs, BBs, or CCBs. All data analyses were performed using SAS 9.4 (SAS Institute, Cary, NC, USA). Two-tailed p values < 0.05 were considered indicative of statistical significance.

**Table 1:** Characteristics of participants stratified by treatment strategy before and after propensity score matching.

Variable	Before propensity score matching			After propensity score matching		
	Thiazide	Non-thiazide	P-value	Thiazide	Non-thiazide	*SMD*
All, n	4192	81,083		4045	4045	
Age (year), mean ± SD	60.2 ± 14.3	51.0 ± 11.7	< 0.0001	59.6 ± 14.1	58.4 ± 12.8	0.09
Sex, n (%)			< 0.0001			
Female	2363 (56.4)	34,420 (42.5)		2265 (56.0)	2223 (55.0)	0.02
Male	1829 (43.6)	46,663 (57.6)		1780 (44.0)	1822 (45.0)	0.02
Duration of hypertension at index (year), mean ± SD	3.45 ± 3.32	0.68 ± 2.00	< 0.0001	3.28 ± 3.19	3.58 ± 4.44	0.08
Comorbidities, n (%)						
Coronary artery disease	1266 (30.2)	12,765 (15.7)	< 0.0001	1167 (28.9)	1178 (29.1)	0.006
Heart failure	556 (13.3)	1121 (1.38)	< 0.0001	442 (10.9)	370 (9.15)	0.06
Stroke	1055 (25.2)	8407 (10.4)	< 0.0001	972 (24.0)	977 (24.2)	0.003
Chronic kidney disease	350 (8.35)	1896(2.34)	< 0.0001	305 (7.54)	297(7.34)	0.008
Diabetes mellitus	1524 (36.4)	21,526 (26.6)	< 0.0001	1444 (35.7)	1448 (35.8)	0.002
Hyperlipidemia	2050 (48.9)	39,272 (48.4)	< 0.0001	1992 (49.3)	2142 (53.0)	0.074
Osteoarthritis	2049 (48.9)	19,941 (24.6)	< 0.0001	1939 (47.9)	1903 (47.1)	0.018
Medications, n (%)						
Antituberculosis agents	84 (2.00)	460 (0.57)	< 0.0001	72 (1.78)	72 (1.78)	0.000
Immunosuppressive agents	39 (0.93)	241 (0.30)	< 0.0001	36 (0.89)	47 (1.16)	0.027
Nicotinic acid	551 (13.1)	5315 (6.56)	< 0.0001	508 (12.6)	566 (14.0)	0.042
Aspirin	1251 (29.8)	17,382 (21.4)	< 0.0001	1187 (29.3)	1218 (30.1)	0.017

*SD* standard deviation, *SMD* standardized mean difference, *ACEIs* angiotensin-converting enzyme inhibitors, *ARBs* angiotensin II receptor blockers, *CCBs* calcium channel blockers, *BBs* beta-blockers.

## Results

### Demographic characteristics of the study cohort

Table 1 encapsulates the baseline characteristics of the study participants, categorized by treatment strategy, and delineated into two sets: pre- and post-propensity score matching outcomes. Initially, a significant discrepancy was noted between the thiazide and non-thiazide groups, with thiazide patients being notably older (mean age: thiazide vs. non-thiazide: 60.2 vs. 51.0 years), having a higher prevalence of females (56.4% vs. 42.5%), and a longer mean duration of hypertension (3.45 vs. 0.68 years). Additionally, the thiazide cohort exhibited a higher rate of comorbidities such as CAD, HF, stroke, CKD, DM, hyperlipidemia, and osteoarthritis. Medications like antituberculosis agents, immunosuppressive agents, nicotinic acid, and aspirin were also more common among thiazide users. Through propensity score matching, the sample was divided into equal-sized groups (n = 4045), effectively reducing initial imbalances, with the standardized mean differences (SMDs) of all variables being significantly low.

### The difference in the prescribing cascade of antigout agents between thiazide and non-thiazide groups

**Figure 2:**
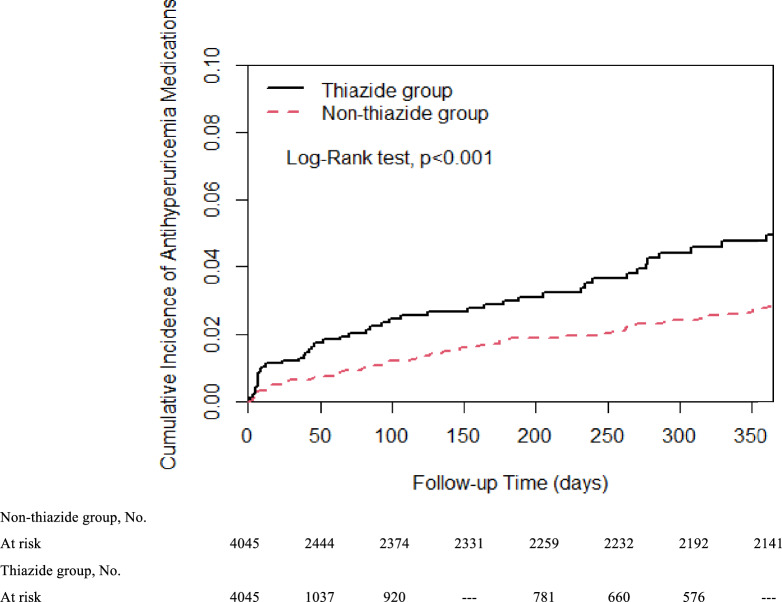
Cumulative incidence of the prescribing cascade of antigout medications between thiazide and non-thiazide groups. Indicates a difference of less than two from the previous data point. The data were withheld by the Taiwan National Health Insurance Registry Database in compliance with privacy protection regulations due to the small number of cases involved.

Individuals who were newly dispensed thiazide diuretics had a higher cumulative incidence of subsequently receiving antigout medications than those who took non-thiazide regimens. The difference started shortly after treatment initiation, and the trend increased for approximately one year (Fig. 2). The crude HR for < 30 days, 31–180 days, and > 180 days were 2.23 (95% CI 1.30–3.85), 2.19 (95% CI 1.21–3.95), and 2.61 (95% CI 1.91–3.56), respectively. Even after adjusting for age, sex, duration of hypertension, comorbidities, concomitant medication, and the index date, the thiazide group showed a higher risk of prescribing cascade with antigout drugs than the non-thiazide group over the three time periods (< 30 days: aHR 2.23, 95% CI 1.23–4.02; 31–180 days: aHR 2.07, 95% CI 1.07–4.00; > 180 days: aHR 2.41, 95% CI 1.73–3.37) (Table 2). An in-depth Cox model analysis was conducted to delineate the risks of prescribing cascades in antigout medication, taking into account the impact of thiazide use, demographic factors, comorbidities, and related medications, with detailed findings of each covariate's effect presented in Supplementary Table S3.

**Table 2:** Relationship between thiazide use and antigout medication prescriptions after propensity score matching in gout-naive adults on first-line antihypertensive therapy.

Comparison	HR (95% CI)		
	1–30 days	31–180 days	> 180 days
Thiazide vs. Non-thiazide			
Crude	2.23 (1.30–3.85)**	2.19 (1.21–3.95)**	2.61 (1.91–3.56)***
Adjusted^a^	2.23 (1.23–4.02)**	2.07 (1.07–4.00)*	2.41 (1.73–3.37)***

*HR* the hazard ratio for time (in days) from the index date *CI* confidence interval.

*p < 0.05; **p < 0.01; ***p < 0.001.

^a^Adjusted for index date, age, sex, duration of hypertension, comorbidities, and concomitant use of medication that might interfere with hyperuricemia.

### Comparison with each class of antihypertension medicine and subgroup analyses

Table 3 presents the association between thiazide diuretic use and the subsequent prescription of antigout medications, contrasting it with the use of other classes of first-line antihypertensive drugs. Compared to those on ACEIs/ARBs, patients using thiazide showed an increased aHR of 2.21 (95% CI 1.17–4.18) within the first 30 days, which decreased slightly to an aHR of 2.01 (95% CI 1.01–4.01) during the 31–180 day interval, and subsequently rose to an aHR of 2.50 (95% CI 1.78–3.50) post 180 days, reflecting the risk pattern found in the entire study cohort as outlined in Table 2. When compared with CCBs and BBs, a statistically significant increase in risk was observed only after 180 days, with aHRs of 2.91 (95% CI 1.55, 5.46) for CCBs and 5.35 (95% CI 1.22, 23.5) for BBs, indicating a trend of increased long-term risk.

**Table 3:** Comparative analysis of thiazide treatment on prescribing cascades of antigout agents in a propensity score matched cohort of gout-naive adults on first-line antihypertensive medicines.

Comparison	Adjusted HR (95% CI)*		
	1–30 days	31–180 days	> 180 days
Thiazide vs. ACEIs/ARBs			
Crude	2.25 (1.25, 4.07)**	2.14 (1.16, 3.95)*	2.55 (1.85, 3.50)***
Adjusted	2.21 (1.17, 4.18)*	2.01 (1.01, 4.01)*	2.50 (1.78, 3.50)***
Thiazide vs. CCBs			
Crude	1.97 (0.70, 5.58)	1.89 (0.70, 5.11)	3.16 (1.82, 5.51)***
Adjusted	2.87 (0.96, 8.63)	1.90 (0.62, 5.85)	2.91 (1.55, 5.46)***
Thiazide vs. BBs			
Crude	2.40 (0.33, 17.5)	–	3.58 (0.87, 14.7)
Adjusted	2.79 (0.36, 21.7)	–	5.35 (1.22, 23.5)*

*HR* hazard ratio, *CI* confidence interval, *ACEIs* angiotensin-converting enzyme inhibitors, *ARBs* angiotensin II receptor blockers, *CCBs* calcium channel blockers, *BBs* beta-blockers.

*p < 0.05; **p < 0.01; ***p < 0.001.

^a^Adjusted for index date, age, sex, duration of hypertension, comorbidities, and concomitant use of medication that might interfere with hyperuricemia; –﻿ the data were withheld by the Taiwan National Health Insurance Registry Database in compliance with privacy protection regulations due to the small number of cases involved.

To determine whether the association between thiazide therapy and the prescription of antigout agents was confined to a specific condition, we performed subgroup analysis disaggregated by sex, age group, and related comorbidities (Table 4). Patients of each sex, age < 50, 50–65, and > 65 years, were all associated with a higher risk of using antigout medicine. The risk was consistently high in the thiazide group regarding comorbidity status, including DM, CKD, CAD, and osteoarthritis. Among them, the interaction was significant between the status of DM and osteoarthritis, which further suggested that the presence of DM and absence of osteoarthritis were associated with a higher risk of prescribing cascades of antigout drugs than their counterparts.

**Table 4:** Comparison of the incidence and hazard ratio of prescribing cascade of antigout medications between thiazide and non-thiazide groups after propensity score matching, stratified by sex, age, and comorbidities.

Variable	Thiazide group			Non-thiazide group				
	Event	PY	Rate^#^	Event	PY	Rate^#^	Crude HR (95% CI)	Adjusted HR^a^ (95% CI)
All	106	679,809	1.56	236	4,774,087	0.49	2.26 (1.76, 2.90)***	2.15 (1.64, 2.82)***
Sex								
Female	35	374,184	0.94	90	2,638,712	0.34	2.08 (1.36, 3.17)***	1.85 (1.17, 2.92)**
Male	71	305,625	2.32	146	2,135,375	0.68	2.40 (1.76, 3.28)***	2.35 (1.68, 3.30)***
*p* for interaction								0.35
Age								
< 50	25	119,802	2.09	56	1,099,575	0.51	2.31 (1.38, 3.87)**	2.75 (1.58, 4.78)***
50–65	32	232,556	1.38	103	2,215,505	0.46	2.31 (1.50, 3.57)***	2.01 (1.28, 3.15)**
> 65	49	327,451	1.50	77	1,459,007	0.53	2.22 (1.50, 3.28)***	2.13 (1.38, 3.31)***
*p* for interaction								0.59
Diabetes mellitus								
No	59	376,095	1.57	157	2,627,341	0.60	1.73 (1.25, 2.39)***	1.49 (1.05, 2.11)*
Yes	47	303,714	1.55	79	2,146,746	0.37	3.68 (2.45, 5.51)***	3.97 (2.53, 6.21)***
*p* for interaction								0.03
Chronic kidney disease								
No	93	595,891	1.56	220	4,310,551	0.51	2.09 (1.60, 2.72)***	1.94 (1.46, 2.59)***
Yes	13	83,918	1.55	16	463,536	0.35	5.68 (2.45, 13.2)***	8.71 (3.15, 24.1)***
*p* for interaction								0.25
Coronary artery disease								
No	68	45,082	1.51	158	3,053,963	0.52	1.86 (1.37, 2.53)***	1.74 (1.25, 2.44)**
Yes	38	229,007	1.66	78	1,720,124	0.45	3.48 (2.25, 5.37)***	3.13 (1.96, 5.02)***
*p* for interaction								0.33
Osteoarthritis								
No	77	352,376	2.19	130	2,437,563	0.53	2.74 (2.02, 3.73)***	2.48 (1.78, 3.45)***
Yes	29	3,427,433	0.89	106	2,336,524	0.45	1.58 (1.01, 2.47)*	1.62 (1.00, 2.61)*
*p* for interaction								0.003

*HR* hazard ratio for time (in days) from index date, *CI* confidence interval, *PY* person-years.

*p < 0.05; **p < 0.01; ***p < 0.001.

^#^Rate: per 10,000 person-years.

^a^Adjusted for index date, age, sex, duration of hypertension, comorbidities, and concomitant use of medication that might interfere with hyperuricemia.

## Discussion

In this population-based retrospective cohort study, new prescription of thiazide diuretics for the management of hypertension in gout-naïve adults was associated with a higher risk of a subsequent prescription of antigout agents compared with those who received non-thiazide regimens of the first-line antihypertensive medications (e.g., ACEIs, ARBs, BBs, CCBs). The association was independent of age, sex, duration of hypertension, comorbidities, and concomitant use of the related drugs. The risk persisted and increased if the thiazide was continued. Upon further comparison with each of the different classes of antihypertensive agents, thiazide use still showed an association with a higher risk for prescribing cascades of antigout agents. In addition, the results were consistent across subgroups of age, sex, or status of related comorbidities.

Our study enrolled 363,457 patients who were followed up until 2017. From the original cohort before propensity score matching, the patients in the thiazide group were older and had a greater proportion of females than those in the non-thiazide group. Previous studies have also shown that people aged > 60 years are more likely to be prescribed thiazide diuretics for hypertension management compared to their younger counterparts, and this trend was found to be more prominent in the female population^23^. Thiazide diuretics have been shown to achieve better blood pressure control in hypertensive patients older than 55 years^24^.

Nonetheless, recent studies have found that thiazide diuretics are usually not the first-choice drug for hypertension management, especially in early-stage hypertension patients^10,16^. In some regions, clinicians tended to add thiazide diuretics when the blood pressure control was suboptimal after using other first-line medication. However, thiazide diuretics were used as the dominant antihypertensive agents in some regions^25^. Thus, thiazide therapy is more likely to be used for older hypertensive patients and those with a long history of hypertension. Similarly, in our cohort, patients who received thiazide regimens had a longer history of hypertension and more comorbidities, including CAD, HF, stroke, CKD, DM, hyperlipidemia, and osteoarthritis. Patients in the thiazide group also had greater odds of being administered medications that might influence the serum uric acid concentration, including antituberculosis agents, immunosuppressive agents, nicotinic acid, and aspirin. Therefore, we used propensity score matching to investigate the correlation between thiazide usage and subsequent prescriptions for antigout agents. The patient characteristics of the two groups were well-balanced with low SMDs.

Adult hypertensive patients treated with thiazide showed a higher risk of a prescribing cascade of antigout drugs during the follow-up interval of < 30 days, 31–180 days, and > 180 days. The cHR increased with time elapsed, from 2.23 to 2.61. After adjusting for potential confounding factors, including the index date, age, sex, duration of hypertension, comorbidities, and concomitant use of medication, the thiazide group was still associated with a higher risk of being added antigout agents across all three observational time intervals (aHR: 2.23, 2.07, and 2.41, respectively). The risk of prescribing cascades of antigout drugs remained two-fold even after 180 days if the thiazide was not discontinued. Our results were in line with a previous study, which showed that the longer the patients take the thiazide diuretics, the higher the incidence of hyperuricemia^23^. Previous research revealed a comparable prescribing cascade of antigout agents after using thiazide in hypertension management, but the association became insignificant after adjusting for comorbidities or baseline urate level^12,26^. The present study demonstrated that the possible unnecessary dispensing of antigout drugs happened within one month and would last even after 180 days if the patients still received thiazide diuretics. Other than patients needing thiazide therapy for better blood pressure control, such as those with resistant hypertension, this avoidable prescribing cascade could be related to the physician’s unawareness or inertia of changing to a non-thiazide regimen. Table 2 illustrates the temporal fluctuations in the risk of prescribing cascades for antigout medications associated with thiazide use. An initial increase in this risk during the first month of treatment could be attributed to the adverse effects of thiazides, potentially resulting in dose adjustments by clinicians or patient non-adherence. This may lead to the subsequent observed dip in risk from 1 to 6 months. However, over the long term, the sustained dose-related impact of thiazides on hyperuricemia seems to elevate the risk of subsequent antigout prescriptions beyond the 6-month mark. Additional research is essential to elucidate the specific mechanisms involved.

We performed a paired comparison to determine the association between antigout drug prescription and the new exposure to the different classes of first-line antihypertensive medications. Many commonly prescribed antihypertensive drugs have been found to impact serum uric acid levels to various degrees; for example, BBs and ARBs have been reported to increase serum uric acid level^17,18^. The present work showed that the risk of adding antigout medicine remained higher in patients receiving a thiazide regimen compared to that in patients receiving all other classes of drugs, i.e., ACEIs/ARBs, CCBs, or BBs.

Subgroup analysis was performed to determine whether the risk differed among different prespecified subgroups. Gout has been observed to be more prevalent in men than in women^21^; however, in our study, both men and women using thiazide showed a higher incidence of dispensing antigout agents compared with thiazide non-users. Raja et al. enrolled 330 hypertensive adults in a randomized cross-section study; they found that thiazide treatment numerically increased serum uric acid levels in women but the difference was not statistically significant^27^. Nonetheless, their study differed from the present work because they included patients with a prior diagnosis of gout and did not provide information on the new prescription of antigout medicine after using thiazide diuretics. Our analysis showed that thiazide treatment was consistently associated with a higher risk of subsequent prescription of antigout drugs across different age groups without a significant intergroup difference. The present work did not observe the additional dangers of prescribing cascades of antigout from new-exposure to thiazide in gout-naïve hypertensive older populations. Since it is difficult to conclude based on the subgroup analysis, further studies might be needed to address these issues in older people.

Uric acid is a potential risk factor for CAD and CKD. Uric acid is believed to induce inflammation and cause endothelial dysfunction, leading to multiple systemic diseases^22^. In our study, irrespective of the presence of these two diseases, the thiazide group was associated with a higher risk of prescribing cascades of antigout drugs. This result further underlines the need to take cognizance of the antigout prescribing cascades from thiazide therapy in hypertensive patients. In previous studies, DM patients were found to have elevated serum uric acid concentrations, which was postulated to be associated with increased insulin resistance that can lead to diminished uric acid clearance^28–30^. Our subgroup analysis revealed that thiazide diuretics were associated with a higher rate of subsequent dispensing of antigout medicine, irrespective of DM status. However, patients with DM might have an even higher risk of prescribing cascades of antigout agents than those without DM (*p* for interaction = 0.01). Further research is required to explore the role of thiazides on uric acid handling in patients with DM.

Osteoarthritis has traditionally been thought of as a degenerative disease. Recent research suggests that osteoarthritis is a complex and inflammatory process in which uric acid in the joints may be a contributing factor. The production of two inflammatory cytokines, IL-18 and IL-1β, is correlated with the presence of uric acid in the synovial fluid; among these, IL-18 was found to correlate with osteoarthritis progression^31^. Although hyperuricemia or gout was not found to have a causal effect on osteoarthritis, many studies have suggested a correlation between osteoarthritis and gout^32,33^. Our subgroup analysis showed the persistence of increased prescribing cascades of antigout drugs irrespective of the presence of osteoarthritis, and patients without osteoarthritis were associated with even higher prescribing cascades than osteoarthritis patients. One of the possible explanations could be that the pre-existing osteoarthritis condition or its medication use may mask the diagnosis of gout^34^.

Hyperuricemia is a well-known complication of cancer treatment, particularly in hematologic malignancies. That is because of the uric acid released from tumor cell lysis, as these patients are treated with serial chemotherapy^35^. Conversely, hyperuricemia was shown to correlate with cancer development and poor prognosis^36,37^. Therefore, we excluded cancer patients for this study because hyperuricemia and the subsequent use of antigout agents are not uncommon in these patients^38,39^.

### Limitations

Owing to some inherent limitations of this study, the results should be interpreted with caution. First, the NHI dataset does not provide lifestyle information or laboratory data, such as serum uric acid, creatinine levels, or BP readings. Potential confounders affecting gout incidence may not have been identified or accounted for during statistical analysis. Therefore, we included hypertensive adults treated with first-line antihypertensive medications to minimize the heterogeneity between the two study groups. Due to a lack of data on baseline serum uric acid, we excluded patients with a diagnosis of gout and using antigout medications in the prior year. Second, since most hypertensive patients receive more than one class of drug and some are dispensed in the form of fixed-dose combinations, it is difficult to explore the impact of any one category of antihypertensive medicine on the prescribing cascade. Our study design thus observed the influence on new exposure to the first-line antihypertensive agents by excluding patients taking any antihypertensive medications in the preceding year. Third, the pharmacological treatment for gout or hyperuricemia includes NSAIDs, steroids, colchicine, and urate-lowering drugs. Depending on the severity, etiology of gout, and comorbidities, one or more kinds of drugs can be used for varying duration to manage a gout attack. Balancing the event detection and the specificity, we defined antigout medications as urate-lowering agents and colchicine. Therefore, some events could be mistakenly recorded as a prescribing cascade for treating gout if the colchicine was used for other indications, such as pericarditis or connective tissue diseases. Since these indications were mostly off-label and the numbers are minimal, the potential impact of this was likely to have been neutralized between the two groups in this population-based study. As for patients who only received NSAIDs or steroids for their gout attacks, the endpoints could be underestimated. However, this situation was more likely to have happened in the thiazide group because physicians would only prescribe NSAIDs or steroids for symptomatic treatment and stop thiazide upon recognition of thiazide diuretics as the temporary trigger for patients’ gout. To reflect real-world prescribing habits more closely, we did not specifically exclude using loop diuretics in the non-thiazide group. This could introduce potential interference from loop diuretics. However, even with this consideration, since loop diuretics are believed to contribute to gout^13,40^, if the thiazide group still shows an increased risk in this comparison, we believe that our overall results remain credible. Last, the data source was from Taiwan NHI, which predominantly comprised Taiwanese people. Therefore, the results should be carefully applied to other ethnic groups.

## Conclusion

Prescription of thiazide diuretics in hypertensive and gout-naïve adults who were newly exposed to the first-line antihypertensive agents was associated with a higher risk of a prescribing cascade of antigout medications compared with those treated with non-thiazide regimens. The risks remained high across the subgroups stratified by age, sex, and related comorbidities and compared with other antihypertensive agents. Physicians should be aware of this avoidable prescribing cascade to prevent unnecessary adverse effects.

### Supplementary Information

Supplementary Tables.

## Data Availability

The data supporting the findings of this study can be obtained from the National Health Insurance Research Database (https://nhird.nhri.edu.tw/). However, the availability of these data is limited as they are used under the permission of the current study, and the original data cannot be downloaded, thus not publicly accessible. Nevertheless, data can be requested independently by the regulations of the National Health Insurance Research Database.

## Abbreviations

ACEIs: Angiotensin-converting enzyme inhibitors
ARBs: Angiotensin II receptor blockers
BBs: Beta-blockers
CAD: Coronary artery disease
CCBs: Calcium channel blockers
CIs: Confidence intervals
CKD: Chronic kidney disease
DM: Diabetes mellitus
HF: Heart failure
HRs: Hazard ratios
ICD-9-CM; ICD-10-CM: International Classification of Diseases, Ninth & Tenth Revision, Clinical Modification
LGTD: Longitudinal generation tracking database
NHI: National health insurance
NHIRD: National health insurance registry database
NSAIDs: Non-steroid anti-inflammatory drugs
