# Supplementary material for: Prescribing cascades of antigout medications from thiazide diuretics in gout-naïve hypertensive adults receiving first-line pharmacological management
Source: Sci Rep. 2024 Mar 28;14:7402. doi: 10.1038/s41598-024-58153-0 (PMC10978838; doi:10.1038/s41598-024-58153-0)
Supplement: Supplementary file 1 — Supplementary Tables. [file 41598_2024_58153_MOESM1_ESM.docx]

**Table S1. Definitions of medications and their ATC codes**

| *At least 1 medication claim* | |
| --- | --- |
| **Medications** | **ATC code** |
| **Antihypertensive agents** |  |
| Thiazide diuretics | C03A |
| Loop diuretics | C03C |
| Angiotensin-converting enzyme inhibitors | C09A, C09B |
| Angiotensin II receptor blockers | C09C, C09D |
| Calcium channel blockers | C08 |
| Beta-blockers | C07 |
| **Antigout agents** | |
| Benzbromarone | M04AB03 |
| Febuxostat, allopurinol | M04AA03, M04AA51 |
| Colchicine | M04AC01 |
| **Medications interfering with uric acid levels** | |
| Antituberculosis agents: pyrazinamide, isoniazide, rifampicin | J04AK01, J04AC01, J04AC51, J04AB02  J04AM |
| Immunosuppressive agents: Cyclosporine | L04AD01 |
| Nicotinic acid | C04AC, C10AD, C10BA01 |
| Acetylsalicylic acid (aspirin) | A01AD05, B01AC06, N02BA01 |

**Table S2. Definitions of diseases and their ICD-9/10 codes**

| **Disease** | **ICD-9-CM** | **ICD-10-CM** |
| --- | --- | --- |
| **Cohort Inclusion** | | |
| Hypertension | 401-405 | I10-I15 |
| **Cohort Exclusions** | | |
| Gout | 274 | M10 |
| Cancer | 140-208 | C00-C96 |
| **Comorbidities** | | |
| Coronary heart disease | 410-414 | I21-I25 |
| Congestive heart failure | 428 | I50 |
| Stroke | 430-438 | I60-I69 |
| Chronic kidney disease | 585, 586 | N18.4-N18.9, N19 |
| Diabetes mellitus | 250 | E08-E13 |
| Hyperlipidemia | 278.00-278.01, 278.1 | E66.09-E66.1, E66.8-E66.9, E66.01, E66.2, E65 |
| Osteoarthritis | 715 | M15-M19 |

**Table S3. Cox Proportional Hazards Regression Analysis of Variables Affecting Antigout Medication Prescription Risk, Including Thiazide Use, Post-Propensity Score Matching**

| Characteristics | Event  (n=342) | PY | Rate^#^ | Crude | Adjusted^a^ |
| --- | --- | --- | --- | --- | --- |
|  |  |  |  | HR (95% CI) | HR (95% CI) |
| Thiazide group | 106 | 679809 | 1.56 | 2.26(1.76, 2.90)*** | 2.15(1.64, 2.81)*** |
| Non-thiazide group | 236 | 4774087 | 0.49 | Ref. | Ref. |
| Sex |  |  |  |  |  |
| Female | 125 | 3012896 | 0.41 | Ref. | Ref. |
| Male | 217 | 2441000 | 0.89 | 2.11(1.69, 2.63)*** | 2.04(1.63, 2.57)*** |
| Age |  |  |  |  |  |
| <50 | 81 | 1219377 | 0.66 | Ref. | Ref. |
| 50–65 | 135 | 2448061 | 0.55 | 0.85(0.64, 1.11) | 1.03(0.78, 1.37) |
| >65 | 126 | 1786458 | 0.71 | 1.04(0.78, 1.37) | 1.29(0.95, 1.75) |
| Diabetes mellitus |  |  |  |  |  |
| No | 216 | 3003436 | 0.72 | Ref. | Ref. |
| Yes | 126 | 2450460 | 0.51 | 0.77(0.62, 0.96)* | 0.88(0.70, 1.11) |
| Chronic kidney disease |  |  |  |  |  |
| No | 313 | 4906442 | 0.64 | Ref. | Ref. |
| Yes | 29 | 547454 | 0.53 | 0.88(0.60, 1.29) | 0.92(0.63, 1.35) |
| Coronary artery disease |  |  |  |  |  |
| No | 226 | 3504765 | 0.64 | Ref. | Ref. |
| Yes | 116 | 1949131 | 0.60 | 0.98(0.78, 1.23) | 1.30(1.01, 1.67)* |
| Osteoarthritis |  |  |  |  |  |
| No | 207 | 2789939 | 0.74 | Ref. | Ref. |
| Yes | 135 | 2663957 | 0.51 | 0.70(0.56, 0.87)** | 0.86(0.68, 1.09) |
| Hyperlipidemia |  |  |  |  |  |
| No | 178 | 2086531 | 0.85 | Ref. | Ref. |
| Yes | 164 | 3367365 | 0.49 | 0.62(0.50, 0.77)*** | 0.72(0.58, 0.91)** |
| Heart failure |  |  |  |  |  |
| No | 297 | 4760274 | 0.62 | Ref. | Ref. |
| Yes | 45 | 693622 | 0.65 | 1.08(0.79, 1.48) | 1.01(0.73, 1.41) |
| Stroke |  |  |  |  |  |
| No | 275 | 3969204 | 0.69 | Ref. | Ref. |
| Yes | 67 | 1484692 | 0.45 | 0.67(0.52, 0.88)** | 0.79(0.59, 1.05) |
| Medications |  |  |  |  |  |
| Antituberculosis agents |  |  |  |  |  |
| No | 339 | 5343107 | 0.63 | Ref. | Ref. |
| Yes | 3 | 110789 | 0.27 | 0.45(0.14, 1.39) | 0.44(0.14, 1.39) |
| Immunosuppressive agents |  |  |  |  |  |
| No |  |  | 0.63 | Ref. | Ref. |
| Yes |  |  | 0.37 | 0.61(0.20, 1.91) | 0.60(0.19, 1.89) |
| Nicotinic acid |  |  |  |  |  |
| No | 316 | 4420657 | 0.71 | Ref. | Ref. |
| Yes | 26 | 1033239 | 0.25 | 0.38(0.26, 0.57)*** | 0.46(0.30, 0.69)*** |
| Aspirin |  |  |  |  |  |
| No | 249 | 3177393 | 0.78 | Ref. | Ref. |
| Yes | 93 | 2276503 | 0.41 | 0.57(0.45, 0.73)*** | 0.59(0.45, 0.78)*** |

Abbreviation: HR, hazard ratio; CI, confidence interval; PY, person-years.

^#^Rate: per 10,000 person-years.

^a^ adjusted for index year, age, sex, comorbidities, treatment, and concomitant medication in Cox proportional hazards regression models.

*p < 0.05; **p < 0.01; ***p < 0.001
